# Serotonin Syndrome Induced Tako-Tsubo Syndrome

**DOI:** 10.1155/2022/7551440

**Published:** 2022-06-26

**Authors:** Ian Lancaster, Jeffrey Steinhoff, Jose Mosco-Guzman, Deep Patel

**Affiliations:** HCA Healthcare/USF Morsani College of Medicine GME/Largo Medical Center, USA

## Abstract

Tako-tsubo syndrome is characterized by temporary systolic dysfunction of the left ventricle in the absence of coronary artery disease. Serotonin syndrome is a life-threatening condition associated with increased serotonergic activity in the central nervous system (CNS). We report a case of Tako-tsubo syndrome following seizures secondary to serotonin syndrome.

## 1. Introduction

Tako-tsubo syndrome is a temporary and reversible systolic dysfunction of the left ventricle's apical area, typically in the setting of an emotional or physical trigger [1]. The exact pathophysiologic mechanism of Tako-tsubo syndrome is unknown with multiple theories postulated including catecholamine-induced cardiotoxicity and microvascular dysfunction [1]. This condition can resemble an acute coronary syndrome (ACS) as both conditions may present with acute onset chest pain, dyspnea, and electrocardiogram changes, most notably acute ST segment elevations [2]. The diagnosis of Tako-tsubo syndrome is challenging in part due to similarities in presentation to an ACS [2]. The most utilized diagnostic criteria used is from the Mayo Clinic and requires that patients have echocardiographic findings of temporary hypokinesis, dyskinesis, or akinesis of the left ventricle; lack of coronary artery disease (CAD); acute electrocardiographic (ECG) changes or elevations in cardiac troponins; and no presence of pheochromocytoma or myocarditis [3]. Once identified, medical therapy with angiotensin-converting enzyme inhibitors and beta blockers is recommended [4].

In rare instances, serotonin syndrome also known as serotonin toxicity is a cause of Tako-tsubo syndrome. It is a potentially life-threating drug-induced toxidrome caused by medications which affect serotonin and ultimately leads to increased serotonergic activity in the peripheral and central nervous systems [5]. Several drug classes have been associated with a condition including selective serotonin reuptake inhibitors, serotonin norepinephrine reuptake inhibitors, serotonin modulators, and tricyclic antidepressants [5]. The clinical presentation of serotonin syndrome is variable with varying symptoms as well as differing levels of symptom severity [6]. The typical triad of clinical features is altered mental status (i.e., agitation and anxiety to confusion), autonomic hyperactivity (i.e., diaphoresis, tachycardia, hyperthermia, and diarrhea), and neuromuscular hyperactivity (i.e., myoclonus, hyperreflexia, and muscle rigidity) [6]. Included within the wide-ranging effects of excess serotonin are arterial vasoconstriction, bronchoconstriction, platelet aggregation, increased GI motility, altered wakefulness, decreased attention, altered affective behavior, altered thermoregulation, increased motor tone, and aggression [7]. Ultimately, this leads to a hyperadrenergic state leading to an increased release of catecholamines [8].

In this case report, we discuss the case of a 26-year-old female who was found to have Tako-tsubo syndrome following a generalized tonic-clonic seizure secondary to serotonin syndrome. We will discuss the proposed pathophysiologic mechanisms, diagnostic criteria, and treatment of Tako-tsubo syndrome.

## 2. Case Presentation

**Figure 1:**
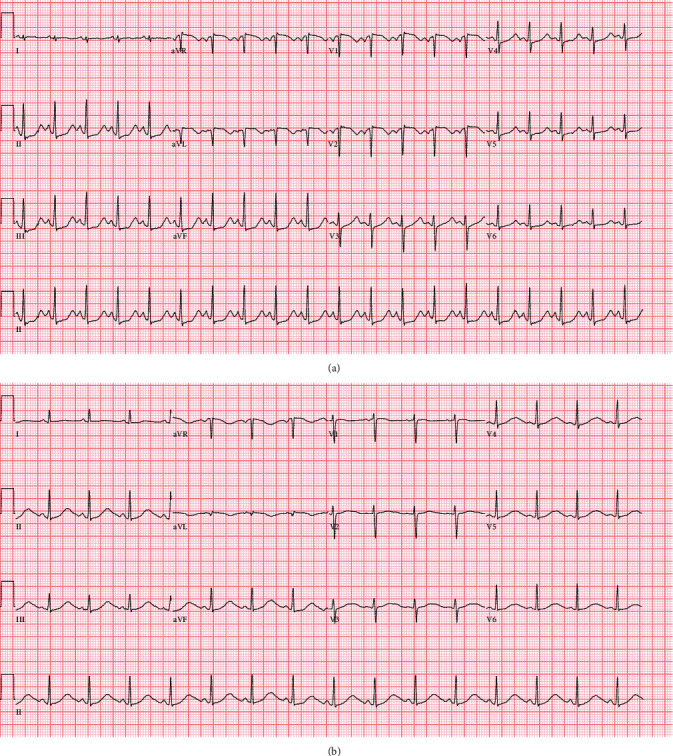
ECG (a): ECG on presentation. Sinus tachycardia with biatrial enlargement, rightward axis deviation, and septal infarction of indeterminate age with diffuse ST segment depressions in leads II, III, aVF, and V3-V6. ECG (b): repeat ECG, 6 hours after admission. Normal sinus rhythm with a prolonged QTc of 631.

A 26-year-old Caucasian female with a past medical history of autism spectrum disorder, epilepsy, and depression presented following a seizure. From the emergency medical services (EMS) report, she had a generalized tonic-clonic seizure of unknown duration shortly before arrival and was encephalopathic during the initial evaluation. She was also noted to have urinary incontinence. While in the emergency department, the ECG showed anteroseptal ST-elevations (Figure 1) and the initial troponin was 1.10 ng/mL. Additionally, the creatine kinase (CK) on admission was 842 units/L. On physical exam, the patient was encephalopathic, myoclonus was present in the ankles, deep tendon reflexes in the upper and lower extremities were noted to be 3+ bilaterally, and the patient had an unremarkable cardiac exam. Cardiology and neurology were consulted, the patient was placed on a heparin drip and given full-dose aspirin, and a STAT transthoracic echocardiogram was ordered.

Reportedly, she was taking fluoxetine and buspirone for her anxiety and depression and zonisamide for her epilepsy as an outpatient. She had been adherent to her recommended psychiatric and antiepileptic regimens.

Additionally, at the time of admission, she was given a loading dose of levetiracetam and her home psychiatric medications were held. In addition to the heparin drip following admission, the troponins were trended and had reached a peak 6 hours after admission of 2.13 ng/mL. The troponin on 12-hour recheck was 1.51 ng/mL Also, her repeat ECG performed 6 hours after admission as well had subsequently changed from anteroseptal ST-segment elevations to normal sinus rhythm with no ST-segment elevations or depressions (Figure 1).

**Figure 2:**
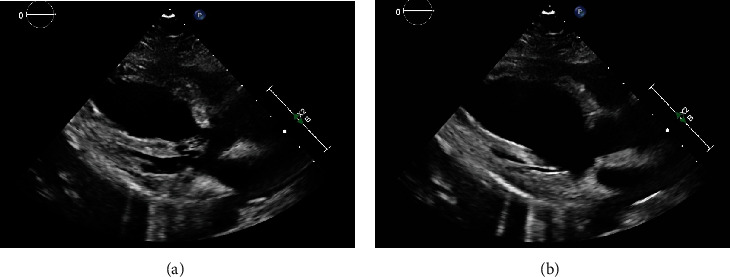
(a) Systole and (b) diastole: transthoracic echocardiogram: left ventricular ejection fraction of 25-30% with reduced systolic function. Akinesis of the basal-mid anteroseptal wall and hypokinesis of the entire inferior wall.

The STAT transthoracic echocardiography demonstrated reduced systolic function of the left ventricle with an ejection fraction of 25-30%. Additionally, the left ventricle was noted to have akinesis of the basal-mid anteroseptal walls and hypokinesis of the inferior wall (Figure 2). There were no abnormalities identified in the right atrium, left atrium, right ventricle, or heart valves.

The day following admission, the patient's encephalopathy had resolved though her CK had continued rise to 1143 units/L. She was continued on the heparin drip until the ischemic evaluation could be completed, her intravenous fluids were continued cautiously given the rising CK, and she was otherwise managed supportively. The patient was then evaluated by neurology who suspected the patient had developed serotonin syndrome due to her home use of fluoxetine and buspirone, acute metabolic encephalopathy, myoclonus, urinary incontinence, and hyperreflexia. As a result, it was recommended that her home psychiatric medications be held indefinitely and levetiracetam be continued for antiepileptic medication.

**Figure 3:**
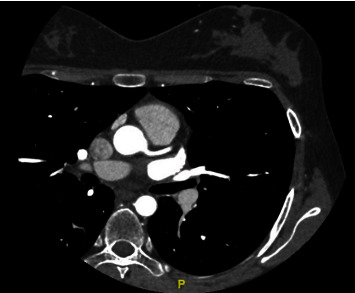
CTA of the coronary vessels demonstrates a patient left main and proximal segment of the left anterior descending branching off of the aorta.

On hospital day 2, the patient's CK had reached its peak of 2155 units/L and later downtrended to 1918 units/L later that day. As her renal function had remained within normal limits with a creatinine of 0.53 mg/dL, a CTA of the coronary vessels was ordered to evaluate for possible ischemic heart disease. The study had demonstrated no significant areas of atherosclerosis or stenosis. No abnormalities of the coronary vessels were identified (Figure 3).

Following the results of the CTA of the coronary vessels, the heparin drip and aspirin were discontinued. Her antiepileptic medication was switched to levetiracetam and her zonisamide. Following continued holding of her psychiatric medication, her myoclonus and hyperreflexia had resolved as well. On hospital day 3, her intravenous fluids were discontinued. Lastly, she was started on metoprolol succinate and lisinopril for treatment of her new nonischemic cardiomyopathy. As she was hemodynamically stable, the patient was deemed appropriate for hospital discharge with instructions to follow up with an outpatient cardiologist in three to four weeks with a repeat echocardiogram to be performed at that time. Lastly, she desired to remain off of anticoagulation at the time of hospital discharge though understood that this would need to be reevaluated as an outpatient.

## 3. Discussion

The exact cause, etiology, and pathophysiologic mechanisms responsible for Tako-tsubo syndrome are unknown. The more accepted theories include catecholamine-induced cardiotoxicity, microvascular dysfunction, and complex integration of neuroendocrine physiology [1]. In most cases, there is believed to be an emotional or physiologic trigger initiating a stress response. Studies have shown that catecholamine concentrations are two to three times higher in Tako-tsubo syndrome in comparison to myocardial infarction. The excessive levels of catecholamines result in intracellular calcium overload consequently leading to myocardial cell injury and dysfunction [9]. This ultimately leads to the development of band necrosis, inflammatory cell infiltration, and fibrosis [10]. The microvascular dysfunction features include the development of endothelium dependent vasodilation, excess vasoconstriction, and abnormal myocardial perfusion which result in myocardial cell apoptosis [11]. In this patient's case of Tako-tsubo syndrome, the source of the excessive catecholamine release is believed to have been due to serotonin syndrome. In serotonin syndrome, the excess release of serotonin can lead to a hyperadrenergic state which may serve as a trigger for Tako-tsubo syndrome [8]. For this patient, her concurrent use of the medications fluoxetine and buspirone likely led to her case of serotonin syndrome.

Upon presentation to the emergency department, not only did the patient have a fever, seizure with postictal confusion, and myoclonus concerning for serotonin; she also had an elevated troponin and anteroseptal ST-segment elevations concerning both ACS and Tako-tsubo syndrome. When considering a case of Tako-tsubo syndrome, the clinical criteria most commonly used to identify a case were developed at Mayo Clinic and consist of four components: (1) temporary hypokinesis, dyskinesis, or akinesis in left ventricular segments with or without apical involvement; aberration in regional wall motion exceeding past a single vascular distribution; and the existence of stress elicitation; (2) the lack of significant coronary artery disease; (3) recent changes detected in the ECG (ST-segment elevation and/or T-wave inversion) or significant elevation of serum cardiac troponins; and (4) nonexistence of pheochromocytoma or myocarditis [3]. In this patient's case, she had anteroseptal ST-segment elevations with a negative coronary CT angiogram, no evidence of a pheochromocytoma or myocarditis, and a left ventricle with akinesis of the basal-mid anteroseptal wall and hypokinesis of the entire inferior wall. To rule out an ACS, the use of coronary vascular imaging, usually with coronary angiography or coronary CT scan, is typically employed to rule out coronary artery disease. Additionally, when the diagnosis of Tako-tsubo syndrome is in doubt, cardiac magnetic resonance imaging can be used to evaluate right ventricular involvement as well as differentiate it from other cardiomyopathies [12]. In this patient's case, her diagnosis of Tako-tsubo syndrome was not in doubt so cardiac MRI was not utilized.

Until an ACS has been ruled out, treatment of Tako-tsubo syndrome typically mimics the treatment of CAD. This typically involves administration of oxygen, heparin, aspirin, and beta blockers [4]. Once Tako-tsubo syndrome is confirmed, it is reasonable to discontinue aspirin and continue beta blocker therapy due to the possible high catecholamine state and angiotensin-converting enzyme inhibitor (ACE-I)/angiotensin receptor blocker (ARB) therapy for regional wall motion abnormality treatment [4]. Anticoagulation can be considered to prevent the development of an apical thrombus and reduce the risk of embolic events [4].

Even though Tako-tsubo syndrome is a transient condition, patients are at a substantial risk of complication, including during the initial hospitalization in which one in five patients is at risk of a significant adverse event [4]. Interestingly, the rates of in-hospital complications for Tako-tsubo syndrome are comparable to patients with an ACS (21% verses 19%) [4]. These adverse events are a result of both acute complications of Tako-tsubo syndrome itself, including cardiogenic shock, and cardiac arrest as well the result of a critical underlying illness [4, 13]. The most common adverse event is hypotension which can vary in severity, and it is usually the consequence of the left ventricular dysfunction [14]. Also of note, left ventricular outflow obstruction can occur and lead to worsening of the apical ballooning as it exposes the cardiac apex to higher wall stress in comparison to the basal myocardium of the left ventricle [15]. Further, both atrial and ventricular arrhythmias may be seen with atrial fibrillation being the most common, approximately 3%, though life-threatening arrhythmias such as torsade de pointes and polymorphic ventricular tachycardia can occur [4, 16]. Lastly, as previously alluded to, systemic thromboembolism is an important complication which can arise and increases the risk of ischemic stroke [2]. The most likely embolic source is from the left ventricle due to stasis of blood due to the wall motion dysfunction leading to the risk of developing a left ventricular mural thrombus [2]. The spontaneous improvement in the left ventricular function can then lead to embolization of the thrombus resulting in the ischemic stroke [2].

The majority of patients have a great prognosis with a recovery rate of 96% [17]. In-hospital mortality rates vary but are typically below 8%, and the recurrence rate is typically under 15% [18]. Usually, left ventricular function begins to recover within days and can fully recuperate within two months [19]. Patients with Tako-tsubo syndrome usually continue on heart failure treatment for twelve months and can be on anticoagulation for the same duration. In this patient's case, she was started on guideline-directed medical therapy with a beta blocker and an ACE-I. She had initially declined anticoagulation though acknowledged that this would be revisited at her follow-up appointment in 1 month with a repeat echocardiogram at that time as well.

## 4. Conclusion

Tako-tsubo syndrome is a temporary and reversible systolic dysfunction of the left ventricle's apical area, typically in the setting of an emotional or physical trigger [1]. In rare instances, this condition may be caused by serotonin syndrome due to the hyperadrenergic state leading to catecholamine release which leads to the development of band necrosis, inflammatory cell infiltration, and fibrosis within cardiac myocytes. Diagnosis of the condition is typically done utilizing the Mayo Clinic Criteria which includes motion abnormalities of the left ventricle, lack coronary artery disease, cardiac troponin or ECG changes, and the nonexistence myocarditis or pheochromocytoma. Initial evaluation of Tako-tsubo includes an ischemic evaluation for rule out of coronary artery disease. Once an ischemic etiology is ruled out, treatment is initiated with a beta blocker and angiotensin-converting enzyme inhibitor or an angiotensin receptor blocker. This treatment should be continued for at least twelve months, and a repeat echocardiogram should be completed 1 month after the identification suspected Tako-tsubo syndrome to ensure recovery of the left ventricular ejection fraction.

## Data Availability

As a case report, the patient's individual information is not available for review, in accordance with HIPPA compliance.
